# Peptide-Grafted Microspheres for Mesenchymal Stem Cell Sorting and Expansion by Selective Adhesion

**DOI:** 10.3389/fbioe.2022.873125

**Published:** 2022-04-12

**Authors:** Shuo Wu, Zongliang Wang, Yu Wang, Min Guo, Mengyang Zhou, Liqiang Wang, Jie Ma, Peibiao Zhang

**Affiliations:** ^1^ School of Pharmaceutical Sciences, Jilin University, Changchun, China; ^2^ Key Laboratory of Polymer Ecomaterials, Changchun Institute of Applied Chemistry, Chinese Academy of Sciences, Changchun, China; ^3^ Department of Ophthalmology, Third Medical Center, Chinese PLA General Hospital, Beijing, China

**Keywords:** surface modification, mesenchymal stem cells, peptide, cell sorting, magnetic microspheres

## Abstract

Mesenchymal stem cells (MSCs) have considerable value in regenerative medicine because of their unique properties such as pluripotency, self-renewal ability, and low immunogenicity. Isolation and purification are prerequisites for various biomedical applications of MSCs, and traditional sorting methods are often expensive, complicated, and difficult to apply on a large scale. In addition to purification, the requirement for expansion of cells also limits the further application of MSCs. The purpose of this study was to develop a unique magnetic sorting microsphere to obtain relatively pure and high-yield MSCs in an economical and effective way, that can also be used for the expansion of MSCs. Poly (ethylene glycol) (PEG)-based anti-adhesive treatment of the prepared oleic acid grafted Fe_3_O_4_-poly (lactic-co-glycolic acid) magnetic microspheres was performed, and then E7 peptide was covalently grafted onto the treated microspheres. Upon a series of characterization, the magnetic microspheres were of uniform size, and cells were unable to adhere to the PEG-treated surface. E7 grafting significantly improved cell adhesion and proliferation. The results obtained from separate culture of various cell types as well as static or dynamic co-culture showed that selective adhesion of MSCs was observed on the magnetic sorting microspheres. Furthermore, the cells expanded on the microspheres maintained their phenotype and typical differentiation potentials. The magnetic properties of the microspheres enabled sampling, distribution, and transfer of cells without the usage of trypsin digestion. And it facilitated the separation of cells and microspheres for harvesting of MSCs after digestion. These findings have promising prospects for MSC research and clinical applications.

## 1 Introduction

Mesenchymal stem cells (MSCs) are fibroblast-like, multipotent, self-renewing adult cells that have been found in various adult tissues, including adipose tissue (Zuk et al., 2001), bone marrow (Pittenger et al., 1999), and umbilical cords (Wang et al., 2004). These cells possess immunomodulatory effects (Yagi et al., 2010), regenerative potential (Zipori, 2004; Picinich et al., 2007), tendency to migrate to site of injury (Sohni and Verfaillie, 2013), and low immunogenicity (Yagi et al., 2010). Because of these inherent properties, MSCs are being researched globally in the context of cell and tissue therapies (including *in vitro* and animal models) to make them therapeutically available for various diseases such as myocardial infarction (Blocki et al., 2015), diabetes (Urban et al., 2008), rheumatoid arthritis (Papadopoulou et al., 2012), acute graft-versus-host disease (Sanchez-Guijo et al., 2014), osteogenesis imperfecta (Gotherstrom et al., 2021), Parkinson’s disease (Meligy et al., 2019), and Alzheimer’s disease (Zhao et al., 2021). Initially, Friedenstein and others developed cultures of MSCs based on their intrinsic physical characteristics that allowed them to attach to the surface of plastic flasks or dishes (Friedenstein et al., 1970). Although this method is time-consuming and only heterogeneous cell populations (Xu et al., 1983; Alhadlaq and Mao, 2004) can be obtained, many laboratories continue to use plastic adhesion methods to isolate MSCs because of the lack of credible purification protocols.

Many techniques have been developed, such as density gradient centrifugation, fluorescent-activated cell sorter (FACS) (Herzenberg et al., 2002), and magnetic-activated cell sorter (MACS) (Schmitz et al., 1994). Although the density gradient centrifugation method enables extraction of cells with a high degree of purity, the first fusion of primary cells takes a long time (Li et al., 2013). FACS and MACS, which are affinity-based methods, also have some limitations such as limited sample throughput and processing speed as well as high operating pressure, which may lead to loss of cell function or vitality; bulky instruments occupying a large number of workbenches; and the requirement for technical expertise to operate complex machinery (Nicodemou et al., 2017). Antibodies, at present, are the mainstay of affinity-based cell purification because of their high capture strength and selectivity. However, antibodies are expensive and generally have low biochemical stability (Ruigrok et al., 2011). In addition, their strong binding ability often makes cell elution challenging (Bacon et al., 2020), and only specific MSCs can be obtained by this method (Nadri and Soleimani, 2007). In recent years, peptides have become a cost-effective and robust substitute for protein ligands (Ruigrok et al., 2011). The E7 peptide (EPLQLKM) has been discovered and used to specifically capture MSCs *in vitro* and *in vivo* due to its strong and specific affinity with MSCs. E7 can bind to human (Shao et al., 2012), rabbit (Meng et al., 2017), and rat (Wu et al., 2019) bone marrow MSCs without species-specificity. The use of poly (ethylene glycol) (PEG) molecules to bind protein ligands and thereby enhance the specificity of the target cells is also coming into view (Yu et al., 2018). Because of its hydrophilic and bioinert properties (Chien et al., 2012; Raos et al., 2018), PEG is considered one of the most widely used and effective antifouling polymers. It also works as an “interlayer” to improve the pliability of the immobilized biomolecules, thus increasing their biological activity (Yu et al., 2011).

The application of MSCs not only requires purity but also raises the need for efficient cell production techniques. Obtaining a sufficient number of cells from source cells requires a large *in vitro* expansion scheme (Kabat et al., 2020). For effective expansion of cells *in vitro*, the method of cultivating cells under a three-dimensional (3D) environment has received increasing attention (Antoni et al., 2015). It can supply a proper spatial environment for cell distribution and provide a natural extracellular matrix (ECM)-simulation environment to enhance the biological activity of cells (Flemming et al., 1999). As carriers for cell multiplication on 3D surfaces, microspheres have proven ideal for cell expansion (Gao et al., 2015; Leong and Wang, 2015), with the following remarkable advantages: 1) simple preparation process; and 2) high specific surface area for maximizing space efficiency and promoting cell attachment and proliferation (Leong and Wang, 2015). Compared with planar culture, microsphere-based culture poses a challenge for harvesting cells. After cell separation, it is necessary to remove the microspheres to obtain a pure cell suspension. Regardless of the method used, the harvesting process involves several complicated steps, which must be carried out in time. A protein-coated microcarrier GEM™ (Global Eukaryotic Microcarrier™, Hamilton, Sweden) (Lin et al., 2014) incorporated with internal magnetic particles has been developed, allowing easy recovery of the beads using magnets. This provides a new method for the preparation and application of microcarriers.

**SCHEME 1:**
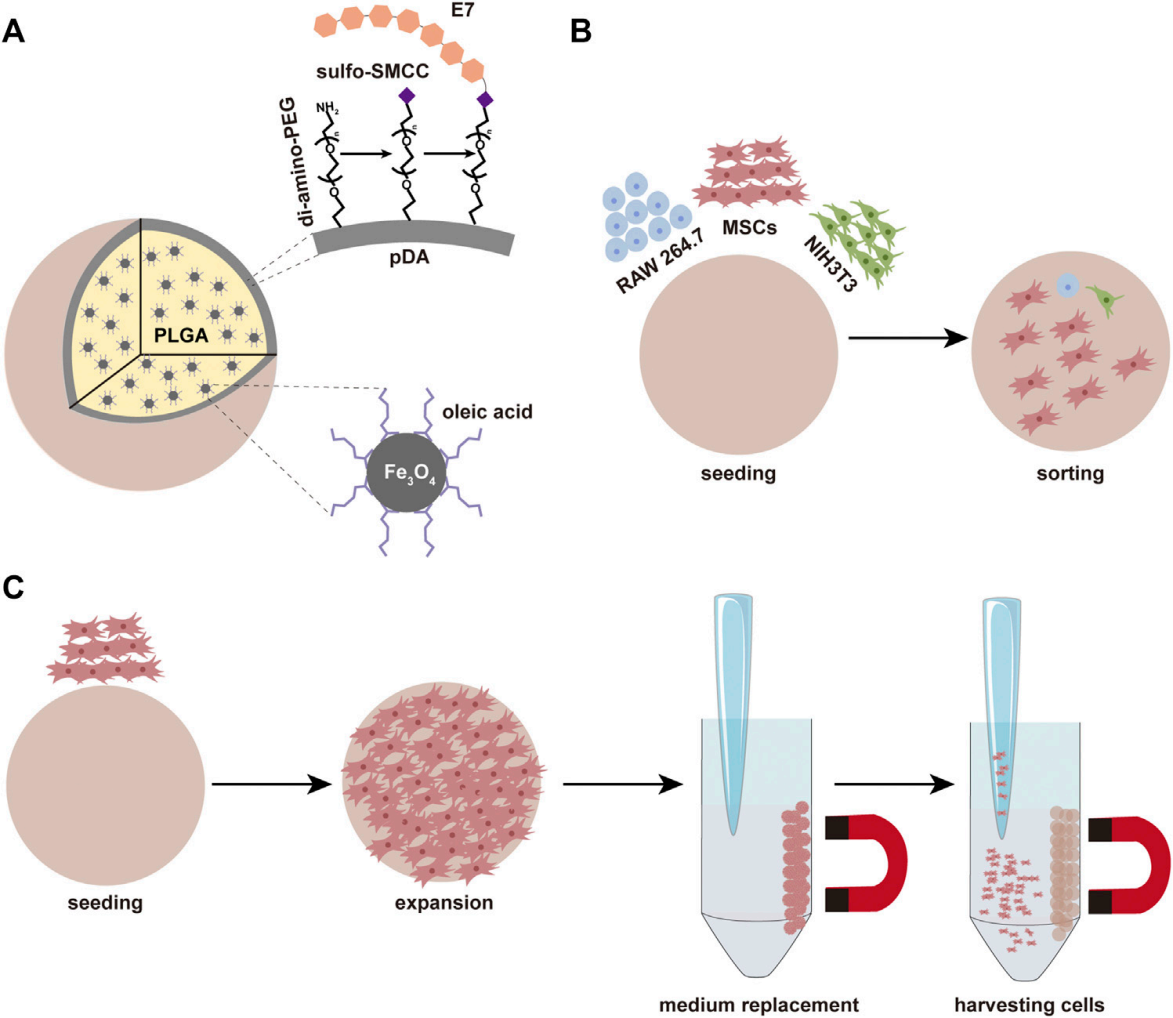
**(A)** The composition of microspheres and the simple process of modification. **(B)** The microspheres can be used for sorting MSCs. **(C)** The microspheres can be used for the expansion of MSCs.

In summary, the most critical challenges in the biological application of MSCs are their isolation and culture. To solve these problems, we doped superparamagnetic nanoparticles (NPs) in polymeric matrix to prepare magnetic microspheres. Moreover, by grafting E7 on the surface of the PEGylated microspheres, selective adhesion of MSCs was achieved, and the proliferation of MSCs on the surface of microspheres was promoted. A range of properties such as specific adhesion efficiency, proliferation, and retention of stemness of MSCs on the magnetic sorting microspheres were also explored (Scheme 1).

## 2 Materials and Methods

### 2.1 Materials and Reagents

All reagents were from Aladdin (Aladdin, China), if not otherwise noted. Poly (lactic-co-glycolic acid) (PLGA, LA:GA = 80:20, *M*w = 200,000) was prepared via ring-opening co-polymerization of L-lactide (LA) and glycolide (GA) in our laboratory (Yan et al., 2019).

### 2.2 Magnetic NP Synthesis

The synthesis of oleic acid grafted Fe_3_O_4_ (Fe_3_O_4_-OA) NPs was slightly modified according to the method previously published by our laboratory (Hao et al., 2019). In brief, iron (III) chloride (10.8 g, 40 mM) and sodium oleate (36.5 g, 120 mM) were blended in a mixture of 60 ml distilled water, 80 ml ethanol, and 140 ml n-hexane, in a round bottom flask. Then, the reaction solution was heated to 70°C for a 4 h reflux with violent magnetic stirring. The brown hexane phase was separated from the solution by cooling and washed three times with 30 ml distilled water. Then, the hexane phase was transferred to a clean round-bottomed flask and dried in a rotary vacuum evaporator with water bath heating. Subsequently, 6.4 ml oleic acid and 253 ml 1-octadecene were added to the red-brown viscous product. The mixing solution was then ultrasonicated for 30 min to complete dissolution. Finally, a glass capillary was added, and slender branch pipes were connected to the mouth of the flask. After holding at 100°C for 1 h, the reaction mixture was heated to 320°C and held again for 1 h. After cooling down to room temperature, the black products were collected using a magnet, washed three times with 100 ml of petroleum ether, and precipitated with 300 ml of ethanol. The obtained product was dried at room temperature for standby application.

### 2.3 Fabrication of Fe_3_O_4_-OA-PLGA Microspheres

Preparation of Fe_3_O_4_-OA-PLGA microspheres was performed using the high-voltage electrostatic (HVE) technique (Yan et al., 2021). Briefly, PLGA (1 g) and Fe_3_O_4_-OA NPs (50 mg) were fully dissolved in dichloromethane (DCM, 10 ml). The solution was loaded into a 2 ml syringe with a 27 G needle. The needle tip and the ethanol replacement solution were linked to the positive and negative electrodes of the HVE droplet generator, respectively. The syringe was advanced at a uniform speed. Microspheres were collected through filtration via sieves and washed successively with ethanol solution and distilled water to remove the excess residual organic solvent.

### 2.4 Modification of Microspheres

To prepare pDA/Fe_3_O_4_-OA-PLGA microspheres, the obtained Fe_3_O_4_-OA-PLGA microspheres were immersed in dopamine solution (2 mg ml^−1^, 10 mM tris, pH = 8.5) for 2 h at room temperature. Then, the pDA/Fe_3_O_4_-OA-PLGA microspheres were incubated in di-amino-PEG (Mw = 2,000, ToYongbio, China) solution (200 mg ml^−1^, 10 mM tris, pH = 8.5) at 40°C for 4 h. The modified microspheres were washed with distilled water and termed PEG/pDA/Fe_3_O_4_-OA-PLGA. In order to enhance the selective adhesion of MSCs, the surface of the microspheres was functionalized with the E7 peptide (GLBiochem, China). The modification of peptide was conducted in the following two ways. 1) The heterobifunctional crosslinker 4-(N-Maleimidomethyl) cyclohexane-1-carboxylic acid 3-sulpho-N-hydroxysuccinimide ester sodium salt (sulfo-SMCC) was used to bind the peptide to the di-amino-PEG molecule on the surface of the microspheres. Sulfo-SMCC was dissolved in distilled water and diluted to 1 mg ml^−1^ with phosphate buffered saline-ethylene diamine tetraacetic acid (PBS-EDTA) coupling buffer. The sulfo-SMCC solution was pipetted into the PEG/pDA/Fe_3_O_4_-OA-PLGA microspheres, followed by incubation for 1 h at room temperature. The microspheres were then covered with E7 peptide solution (2 mg ml^−1^, coupling buffer) and incubated overnight at 4°C to obtain E7/PEG/pDA/Fe_3_O_4_-OA-PLGA. 2) For E7/pDA/Fe_3_O_4_-OA-PLGA microspheres, pDA/Fe_3_O_4_-OA-PLGA microspheres were incubated with E7 peptide solution (2 mg ml^−1^, 10 mM tris, pH = 8.5) overnight at 4°C. If used for cell experiments, the above processes were completed under sterile conditions after the alcohol disinfection of Fe_3_O_4_-OA-PLGA microspheres. Partial microspheres were dried under vacuum for further characterization.

### 2.5 Characterization

The chemical structure of Fe_3_O_4_-OA NPs and PEG-modified microspheres was determined by a Perkin Elmer 2000 FTIR instrument (Perkin Elmer, Germany). A Tecnai G2S-Twin transmission electron microscope (FEI, United States) was used to record the size and shape of the Fe_3_O_4_-OA NPs. The magnetic properties were determined in a Quantum Design-MPMS-XL7 vibrating sample magnetometer (VSM) system (United States) at 300 K. A Philips XL30 ESEM-FEG instrument (Philips, Japan) was used to observe the surface morphology of microspheres. Elemental mapping was carried out by an XL-30W/TMP scanning electron microscope (Philips, Japan). Static water contact angles were measured using a VCA 2000 contact angle system (AST, United States). The percentage of grafting was measured using a TGA500 thermogravimetry analyzer (TA Instruments, United States). Each sample was heated from room temperature to 600°C at 10°C min^−1^.

### 2.6 Cell Isolation and Culture

MSCs were isolated from bone marrow of the tibias and femurs of Sprague-Dawley (SD) rats (100 g, male), as reported previously (Soleimani and Nadri, 2009). The experimental protocol was approved by the Institutional Animal Care and Use Committee of Jilin University School of Pharmaceutical Science. The extracted cells were seeded in a 100-mm cell culture dish (Nest, China) and placed in a humidified incubator at 37°C with 5% CO_2_. The culture medium was changed every 3 days until the cells reached 80% confluency for subculture. All experiments were carried out during passages 3–5. NIH3T3 and RAW 264.7 cell lines were purchased from ATCC and cultured under standard conditions.

### 2.7 Proliferation and Morphology of MSCs on Microspheres

MSCs were seeded at a density of 2 × 10^4^ cells well^−1^ in 48-well (containing 30 µl microspheres) for 1, 3, and 7 days. At each predetermined time point, 500 μl of fresh medium containing 30 μl of CCK-8 (Solarbio, China) was used to replace the medium, and the plate was incubated for 2 h at 37°C. After incubation, the solution (100 μl) in each well was pipetted into a new 96-well plate and measured the absorbance at 450 nm using an Infinite M 200 microplate reader (Tecan, Switzerland). For observing the morphology of cells seeded on each microsphere, double staining of 1,1′-dioctadecyl-3,3,3′,3′tetramethylindocarbocyanine perchlorate (DiI) (Meilun Biological, China) and 4′,6-diamidino-2-phenylindole dihydrochloride (DAPI) (Invitrogen, United States) was performed after 3 days. In order to assess the cell area fraction on various microspheres, the image analysis software ImageJ was used.

### 2.8 Anti-Cell Adhesion Properties of PEG-Modified Microspheres

In order to evaluate the cell-adhesion-resistant effect of PEG/pDA/Fe_3_O_4_-OA-PLGA microspheres, NIH3T3 cells at a density of 2 × 10^4^ cells well^−1^ were seeded on microspheres in a 48-well plate and cultured for 12 h. Cell adhesion was observed by fluorescence microscopy with DAPI staining.

### 2.9 Selective Adhesion of Separately Cultured Multiple Cells

MSCs, NIH3T3, and RAW 264.7 cells were seeded on the different microspheres in 48-well plates at a density of 2 × 10^4^ cells per well. After incubation for 6 h, the microspheres were washed with PBS, respectively stained with DiI, Calcein-AM (Sigma, Germany), and Hoechst 33342 (Beyotime, China), and viewed under a fluorescent microscope.

### 2.10 Selective Adhesion of Co-Cultured Multiple Cells

#### 2.10.1 Static Culture

According to the manufacturer’s instructions, MSCs, NIH3T3, and RAW 264.7 cells were stained with DiI, Calcein-AM and Hoechst 33342, respectively, and co-incubated with a seeding density of 2 × 10^4^ in wells for 6 h. Non-adherent cells were washed away with PBS, and the proportion of each cell type was determined by fluorescence microscopy.

All other experimental conditions remained unchanged, except that RAW 264.7 cells were subjected to Calcein-AM staining, and the cells were co-cultured for 12 h to observe their morphology on the microspheres.

#### 2.10.2 Dynamic Culture

Then, 1.5 ml of the same mixed cell suspension was added to a 5 ml-EP (containing 250 µl microspheres) tube at the same density as described above. The EP tube was placed in home-made equipment (Supplementary Figure S1) by our laboratory. After blending, the tube was allowed to stand for 40 min and rotated for 10 min at 50 rpm with direction changing every second for 4 h, at 37°C. The proportions of different cells were observed and calculated.

### 2.11 Stemness of the Adherent MSCs

MSCs cultured on E7/PEG/pDA/Fe_3_O_4_-OA-PLGA microspheres for 7 days were digested by trypsin digestion for subsequent experiments. An appropriate amount of cells was used to prepare a single-cell suspension, and monoclonal antibodies CD29, CD90, and CD34 (Invitrogen, United States) were added separately to each tube. After incubation at room temperature for 30 min in the dark, the cells were washed with PBS three times to remove the unbound antibodies and detected by flow cytometry. Osteogenic, adipogenic (Puhebio, China), and chondrogenic (Cyagen, China) induction mediums were used instead of basic medium for culture, respectively, to evaluate the differentiation potential of the expanded MSCs. To confirm osteogenesis, cells were stained with the alkaline phosphatase (ALP) assay kit on day 3 of culture and alizarin red on day 14 of culture. Adipogenic differentiation was detected by Oil Red O staining on the 21st day. Alcian blue staining was used to detect the formation of cartilage following 28 days.

### 2.12 Statistical Analysis

Statistical analysis was conducted using analysis of variance (ANOVA, one-way, Origin 8.0, United States). The data were presented as means ± standard deviations (SD). In each experiment, triplicate samples were evaluated. Significant differences were defined at *p* < 0.05.

## 3 Results and Discussion

### 3.1 Characterization of Magnetic NPs

**FIGURE 1:**
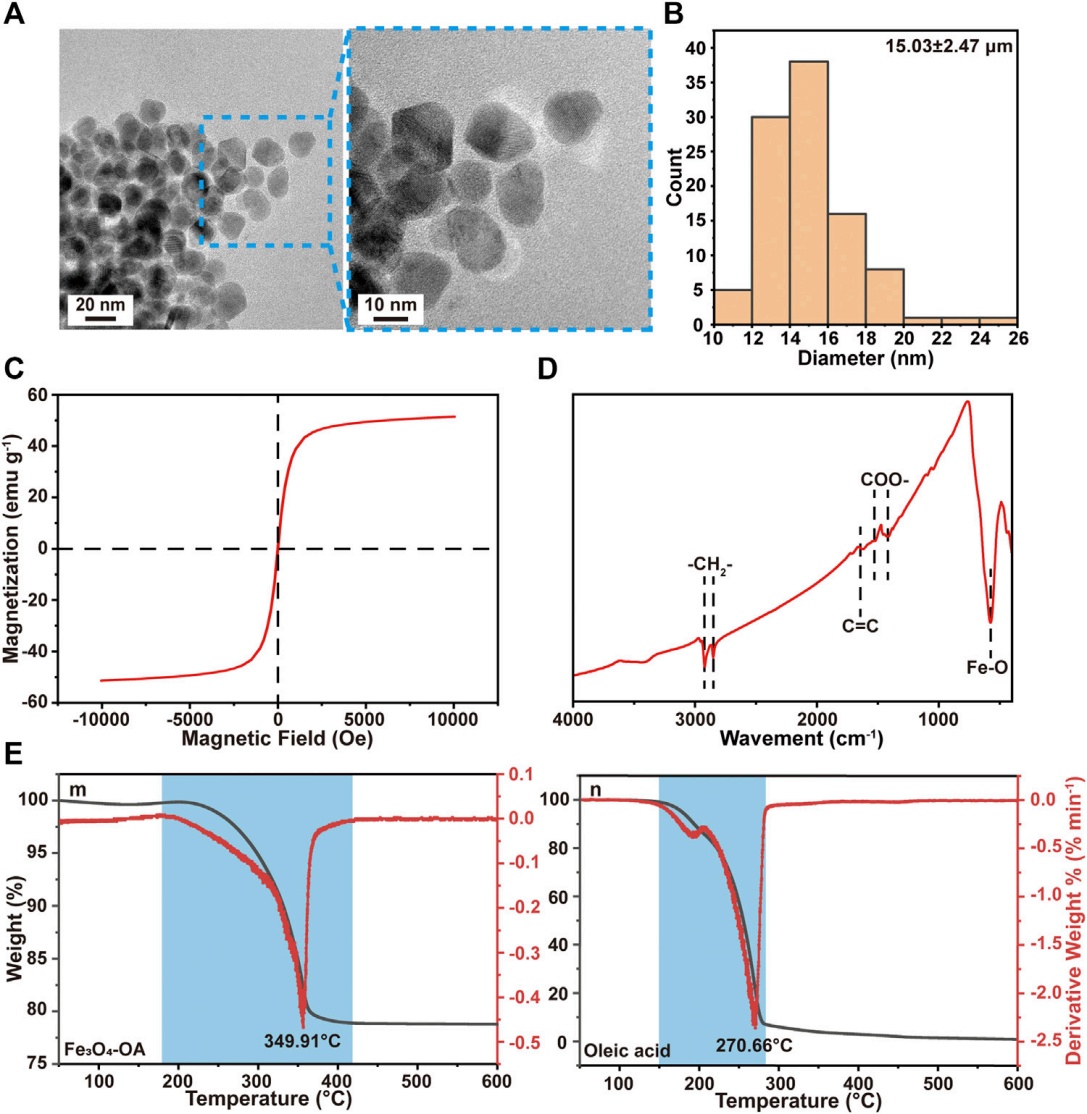
TEM images **(A)** of Fe_3_O_4_-OA NPs at different magnifications and particle size distribution **(B)** of Fe_3_O_4_-OA NPs. VSM curve **(C)** of Fe_3_O_4_-OA NPs. The FTIR spectra **(D)** of Fe_3_O_4_-OA NPs. TGA and DTA curves **(E)** of Fe_3_O_4_-OA NPs (m) and oleic acid (n).

The dispersion stability of Fe_3_O_4_-OA NPs was first examined in chloroform. After standing for 50 h, Fe_3_O_4_ NPs (Supplementary Figure S2A) were precipitated, while Fe_3_O_4_-OA NPs (Supplementary Figure S2B) remained well dispersed: given that Fe_3_O_4_ NPs are poorly dispersed in organic solvents, this was probably due to magnetostatic (magnet dipole-dipole) interactions between magnetic particles (Maity and Agrawal, 2007). However, the mutual repulsion of OA groups on the surface of Fe_3_O_4_-OA NPs can weaken this phenomenon. This facilitated the preparation of homogeneous magnetic composites. When a magnet was applied, Fe_3_O_4_-OA NPs were gathered to the side close to the magnet (Supplementary Figure S2C), indicating that they had good magnetic responsiveness. Based on these findings, it could be preliminarily concluded that Fe_3_O_4_-OA NPs were successfully synthesized. Fe_3_O_4_-OA NPs were characterized using TEM. Figure 1A shows TEM images of Fe_3_O_4_-OA NPs, where it can be seen that the NPs were polygonal and homogeneous in size. TEM images were further analyzed by ImageJ, in which at least 100 NPs were measured (Figure 1B). The average size of the NPs was about 15.03 ± 2.47 nm. The size of the individual iron oxide NPs was typical of single domain superparamagnetic iron oxides (Teja et al., 2009), as verified in subsequent magnetic measurements. The magnetization versus the applied field curves was shown in Figure 1C. At T = 300 K, neither remanence nor coercivity could be detected for the sample with saturation magnetization of 51.4 emu g^−1^, indicating that superparamagnetic behavior existed. Superparamagnetic iron oxide nanoparticles are of increasing interest in the biomedical field due to their unique small size, physical properties, low toxicity, and biocompatibility (Neuberger et al., 2005; Hao et al., 2021). As shown in Figure 1D, stretching vibration of the Fe-O bonds located at about 578 cm^−1^ was observed in the spectra. In the spectrum of Fe_3_O_4_-OA NPs, the peak at 2,849 cm^−1^ was due to the symmetrical stretching vibration of methylene groups, while the peak at 2,920 cm^−1^ was attributed to the asymmetric stretching vibration of methylene groups. These bands are a trait of unsaturated hydrocarbon chains of OA. It was found that the two peaks of the sample were concentrated at 1,512 cm^−1^ and 1,417 cm^−1^, which corresponded to the vibration modes of asymmetric and symmetric carboxylate groups. The stretching vibration of the carbon-carbon double bond in sodium oleate was responsible for the peak at 1,656 cm^−1^. This indicated that the carboxyl group of oleic acid was chemisorbed on the surface. The weight loss shown by the TGA curve indicated that the grafting of OA on Fe_3_O_4_-OA NPs was 21.247 wt.%. The peak of the DTA curve for oleic acid in Figure 1E was at 270.66°C, while the peak of the DTA curve for Fe_3_O_4_-OA was at 349.91°C. This weight loss was related to the decomposition of OA covalently bonded to the surface of the NPs (Mahdavi et al., 2013).

### 3.2 Characterization of Microspheres

**FIGURE 2:**
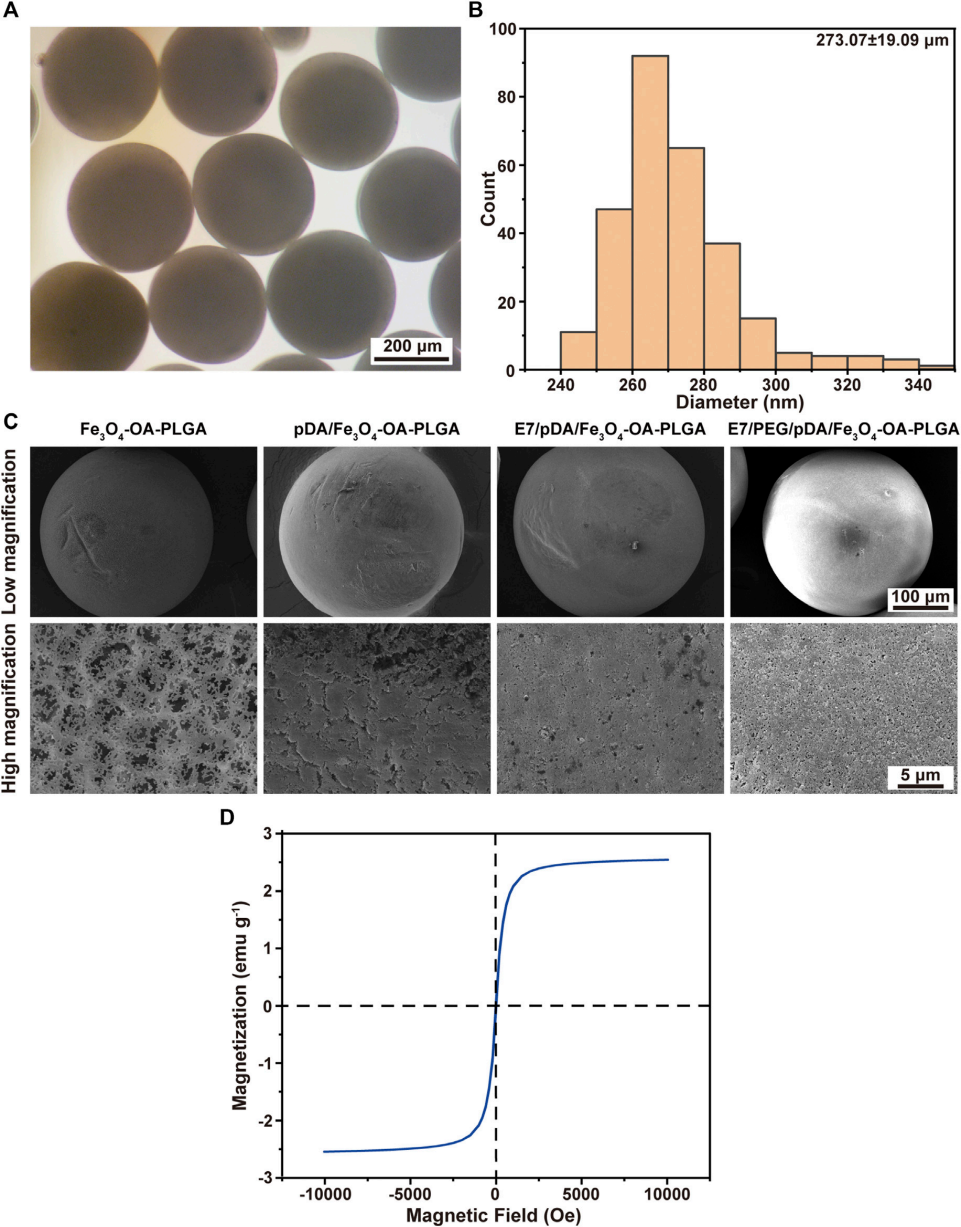
Micrographs **(A)** and size distribution **(B)** of Fe_3_O_4_-OA-PLGA microspheres. SEM images **(C)** of microspheres under low (above) and high (below) magnification. VSM curve **(D)** of Fe_3_O_4_-OA-PLGA microspheres.

Macroscopic observation showed that the Fe_3_O_4_-OA-PLGA microspheres (right) were darker in color than the Fe_3_O_4_-PLGA microspheres (left) (Supplementary Figure S3A). This was caused by the presence of oleic acid on the surface of Fe_3_O_4_-OA NPs. The alkane chains on the surface of Fe_3_O_4_-OA NPs improved the interfacial compatibility with the hydrophobic polymer matrix (Okassa et al., 2007; Hao et al., 2019). Therefore, the dispersion of Fe_3_O_4_-OA NPs in PLGA solution was more uniform than that of Fe_3_O_4_ NPs, and the NPs were not easy to precipitate and clog the needle. As seen from the optical microscope, these microspheres were round and regular (Figure 2A), with a diameter of about 273.07 ± 19.09 μm (Figure 2B). The surface morphology of different microspheres was observed by SEM (Figure 2C). Because they were formed by solvent extraction, the microspheres had well-formed sphericity and a porous surface. After pDA modification, the microspheres acquired a rough surface, although their spherical shape was preserved. The saturation magnetization was 2.5 emu g^−1^ for microspheres (Figure 2D). The magnetic field had a significant effect on the distribution of microspheres: in the absence of a magnet (Supplementary Figure S3B), the microspheres in the water gradually sank because of gravity. After applying the magnet (Supplementary Figure S3C), the microspheres were quickly attracted to the vial wall near the magnet.

### 3.3 Characterization of PEGylated Microspheres

PEG is a versatile polymer that is non-toxic and FDA-approved for use in biomedical research applications, including tissue engineering and drug discovery (Raina et al., 2021). Because of the cell-repulsive and non-adhesive properties of PEG, di-amino-PEG is used here as a microsphere surface modifier for facile conjugation and subsequent activation of PEG molecules on the microsphere surface. PEG can act as an anti-adhesive barrier or binding to peptide to promote cell adhesion. Cells cannot adhere to PEG, which helps facilitate contact between the cells and the peptide.

**FIGURE 3:**
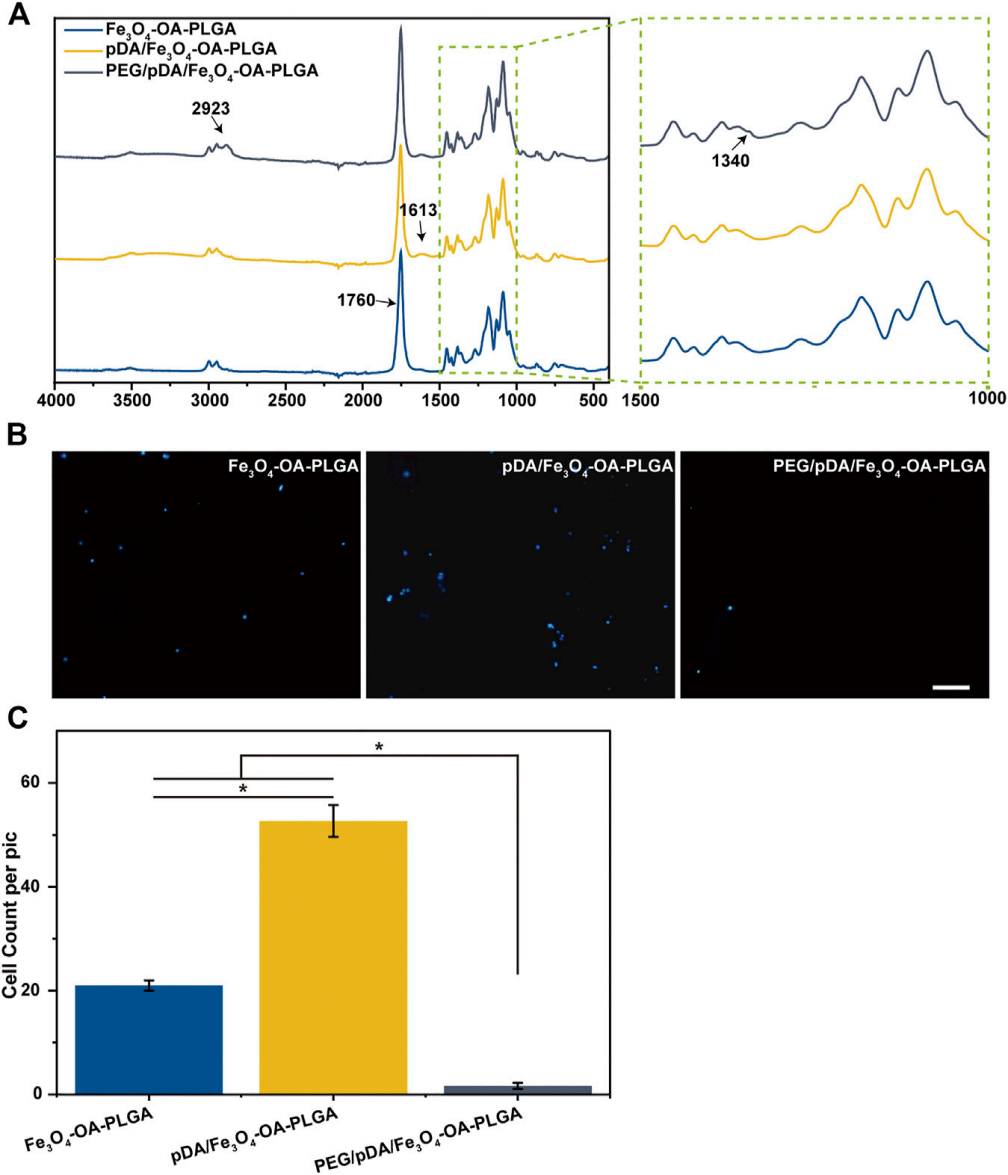
FTIR spectrum **(A)**. Fluorescent images **(B)** of NIH3T3 cells cultured on microspheres for 12 h, and cell count data **(C)**. The cells were stained with DAPI (nucleus, blue). Scale bar = 100 μm. *p* < 0.05, *n* = 3.

Figure 3A shows the FTIR spectra of the microspheres after dopamine coating and PEG grafting modification. The strong signal at 1760 cm^−1^ that appeared in all spectra could be attributed to the presence of carbonyl groups in the PLGA. The N-H bending vibration peak at 1,613 cm^−1^ after dopamine coating, which might be associated with the amino group contained in polydopamine (pDA), could be seen in the spectra. This indicated the polymerization reaction of dopamine on the surface of the microspheres to form the pDA coating layer. From the spectra, it could be observed that the peak of 2,923 cm^−1^ appeared after grafting PEG on the surface of pDA coated microspheres, suggesting that the tensile vibration of C-H was enhanced. The signal at 1,340 cm^−1^ revealed the strengthening of the C-H bending vibration. This implied that PEG had been grafted on the surface of pDA-coated microspheres.

The NIH3T3 cells were inoculated on the surface of the Fe_3_O_4_-OA-PLGA, pDA/Fe_3_O_4_-OA-PLGA, and PEG/pDA/Fe_3_O_4_-OA-PLGA microspheres. After incubation for 12 h, the adherent cells were fixed and stained with DAPI nuclear dye, then observed and statistically analyzed. Fluorescence photographs (Figure 3B) showed that NIH3T3 cells could adhere to the pDA/Fe_3_O_4_-OA-PLGA and Fe_3_O_4_-OA-PLGA microspheres, but they could not adhere well on the PEG/pDA/Fe_3_O_4_-OA-PLGA microspheres. Statistical analysis (Figure 3C) showed that cell adhesion on the PEG/pDA/Fe_3_O_4_-OA-PLGA microspheres was significantly lower than that on the pDA-modified and control surfaces. These results suggest that PEG molecules grafted on the microspheres formed a surface that was more resistant to cell adhesion than the untreated microspheres.

### 3.4 Characterization of Peptide-Modified Microspheres

**FIGURE 4:**
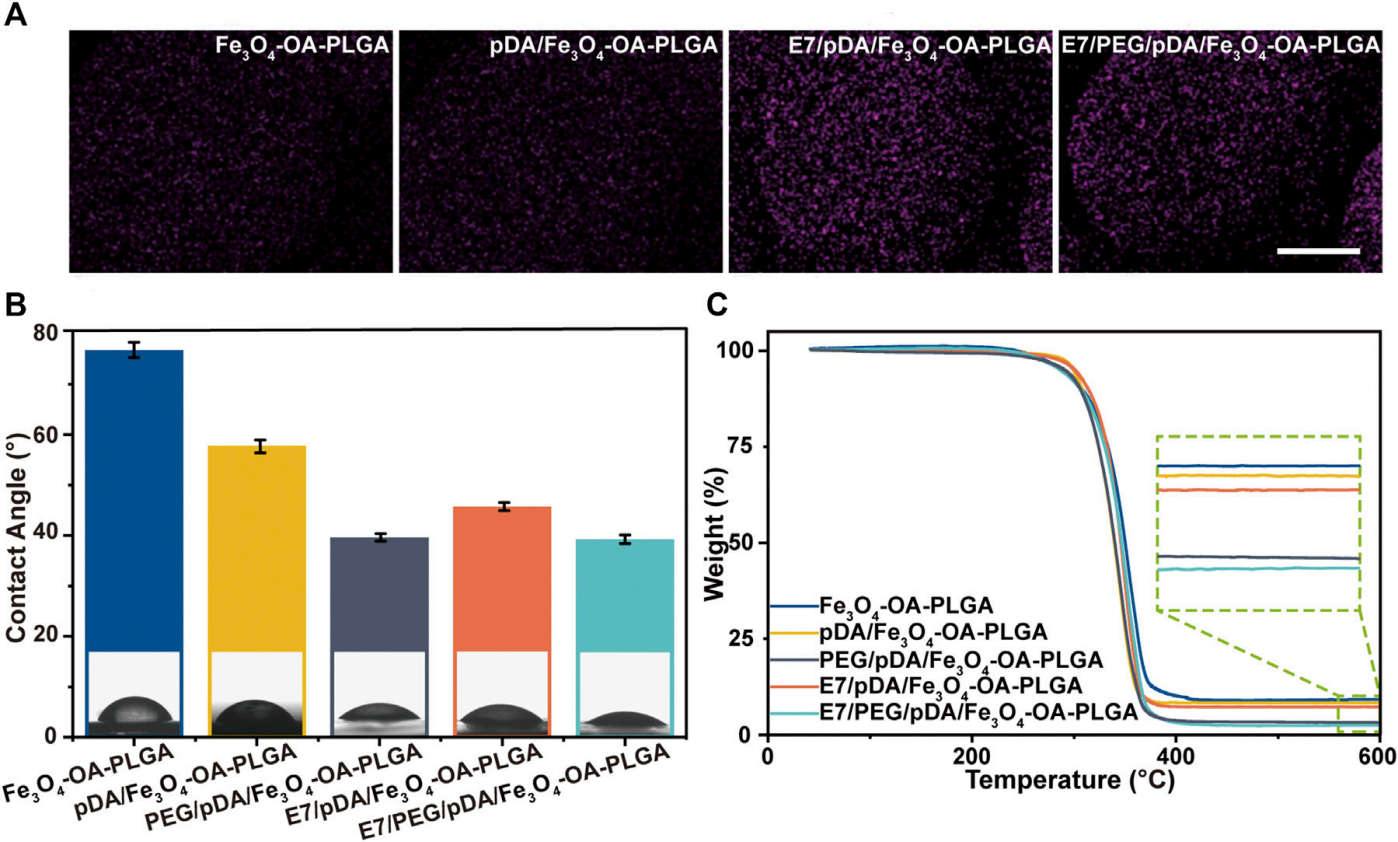
Elemental mapping of sulfur **(A)**. Scale bar = 100 μm. Water contact angle of various microspheres **(B)**. TGA curves of different microspheres **(C)**. *p* < 0.05, *n* = 3.

In this study, selective adhesion to MSCs was achieved by grafting E7 peptide on PEGylated magnetic microspheres. The activation of grafted di-amino-PEG molecules on the microsphere surface depended on their free terminal amine groups. First, we attached sulfo-SMCC to the di-amino-PEG molecule on the surface of the microspheres. Then, the E7 peptide was ligated to the sulfo-SMCC molecule on the surface of the PEG/pDA/Fe_3_O_4_-OA-PLGA microspheres via the Cys residue at the C-terminal end of the peptide. The E7/pDA/Fe_3_O_4_-OA-PLGA microspheres were modified with E7 by pDA coating. Since only the peptide contained sulfur elements, the mapping of sulfur elements was used to characterize the grafting of the peptide. As seen in Figure 4A, the surfaces of E7/pDA/Fe_3_O_4_-OA-PLGA microspheres and E7/PEG/pDA/Fe_3_O_4_-OA-PLGA microspheres were uniformly covered with elemental sulfur. This result indicated that the E7 peptide was successfully immobilized on the magnetic microspheres.

The water contact angle was 79.76 ± 1.52° for Fe_3_O_4_-OA-PLGA microspheres, 57.46 ± 1.27° for pDA/Fe_3_O_4_-OA-PLGA microspheres, and 45.79 ± 0.81° for E7/pDA/Fe_3_O_4_-OA-PLGA microspheres (Figure 4B). Applying pDA and E7 to the microsphere surface decreased the water contact angle, indicating that they improved the hydrophilicity. This was also expected, because pDA and E7 comprise many hydrophilic functional groups, such as amino and hydroxyl groups. The surface of PEG/pDA/Fe_3_O_4_-OA-PLGA and E7/PEG/pDA/Fe_3_O_4_-OA-PLGA microspheres became more hydrophilic, with a contact angle of only about 39.13 ± 0.74° and 39.26 ± 0.87°, possibly due to the presence of PEG with excellent hydrophilicity on the modified surface (Lee et al., 1995).

The organic coating content on the prepared microsphere surface was calculated using the TGA method (Figure 4C). The mass loss values for Fe_3_O_4_-OA-PLGA, pDA/Fe_3_O_4_-OA-PLGA, PEG/pDA/Fe_3_O_4_-OA-PLGA, E7/pDA/Fe_3_O_4_-OA-PLGA, and E7/PEG/pDA/Fe_3_O_4_-OA-PLGA were at 91.038, 91.858, 97.021, 92.957, and 97.725 wt.%, respectively. E7 grafting of E7/pDA/Fe_3_O_4_-OA-PLGA and E7/PEG/pDA/Fe_3_O_4_-OA-PLGA was calculated to be 1.099 wt.% and 0.704 wt.%, respectively, which might be related to the coupling efficiency of sulfo-SMCC.

### 3.5 Evaluation of the Cytocompatibility of Microspheres

Because cells could not adhere to the PEG/pDA/Fe_3_O_4_-OA-PLGA microspheres, the PEG/pDA/Fe_3_O_4_-OA-PLGA microspheres were not evaluated in subsequent experiments.

#### 3.5.1 Cytotoxicity

The toxicity of the microsphere extracts was assessed first (Supplementary Figure S4). For the 100% extract, the cell viability ranged from 87.64 ± 2.14% to 94.33 ± 2.96%. For the 50% extract, the cell viability ranged from 91.36 ± 2.033% to 97.32 ± 2.99%. Indirect cytotoxicity assays indicated that these microspheres were safe for *in vivo* application. Although nanoparticles were used, the effect on cell viability was negligible. The binding ability between Fe_3_O_4_-OA NPs and PLGA matrix might have been strengthened by the oleic acid modification layer on the magnetic nanoparticles, thus reducing their leaching from the microspheres (Hao et al., 2019). In addition, after washing, reagents harmful to the cells were reduced to a reasonable level.

#### 3.5.2 Cell Proliferation

**FIGURE 5:**
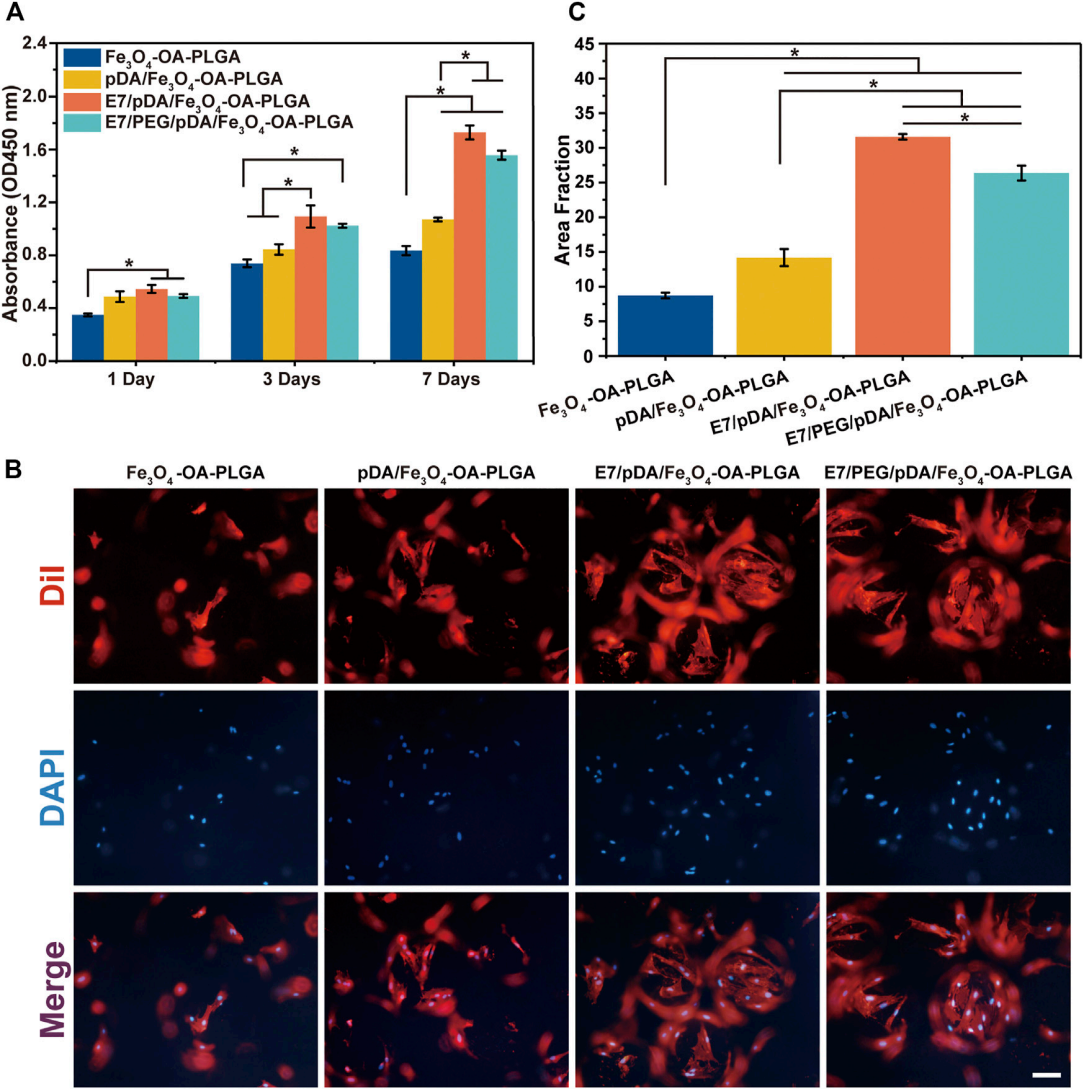
Proliferation of MSCs on microspheres at 1, 3, and 7 days post-seeding evaluated by CCK-8 assay **(A)**. The cellular morphology **(B)** and statistical area fraction **(C)** of MSCs cultured for 3 days on the different microspheres, which were stained by DiI (cytoplasm, red)/DAPI (nuclei, blue). Scale bar = 100 μm. *p* < 0.05, *n* = 3.

Microspheres should support cell attachment and proliferation. To assess cell behavior, MSCs were seeded and cultured on the microspheres. Cell proliferation was detected on days 1, 3, and 7 with the CCK-8 kit (Figure 5A). According to the results, the cell viability of the three groups of microspheres was similar on the first day except for the Fe_3_O_4_-OA-PLGA microspheres. After 3 and 7 days, E7/pDA/Fe_3_O_4_-OA-PLGA and E7/PEG/pDA/Fe_3_O_4_-OA-PLGA exhibited higher cell viability. These results corresponded to those for TGA. E7 grafting affected cell adhesion and proliferation, thus showing better biocompatibility of the microspheres, because E7 is an MSC-specific affinity peptide with a superior affinity for MSCs.

#### 3.5.3 Cell Morphology

Fluorescence microscopy was used after 3 days to assess the distribution of MSCs on the microspheres (Figure 5B). In the presence of E7 peptide, the number of cells adhering to the surface of E7/pDA/Fe_3_O_4_-OA-PLGA and E7/PEG/pDA/Fe_3_O_4_-OA-PLGA microspheres was increased, and the area fraction (Figure 5C) was significantly higher compared with that of the pDA/Fe_3_O_4_-OA-PLGA and Fe_3_O_4_-OA-PLGA groups. Cells were uniformly and densely distributed on the E7/pDA/Fe_3_O_4_-OA-PLGA and E7/PEG/pDA/Fe_3_O_4_-OA-PLGA microspheres, showing excellent cell adhesion and spreading. These results indicate that the functionalization of grafted di-amino-PEG molecules with E7 peptide not only restored cell adhesion to the surface but also enhanced it relative to the Fe_3_O_4_-OA-PLGA and pDA/Fe_3_O_4_-OA-PLGA.

### 3.6 Selective Adhesion of Separately Cultured Multiple Cells

**FIGURE 6:**
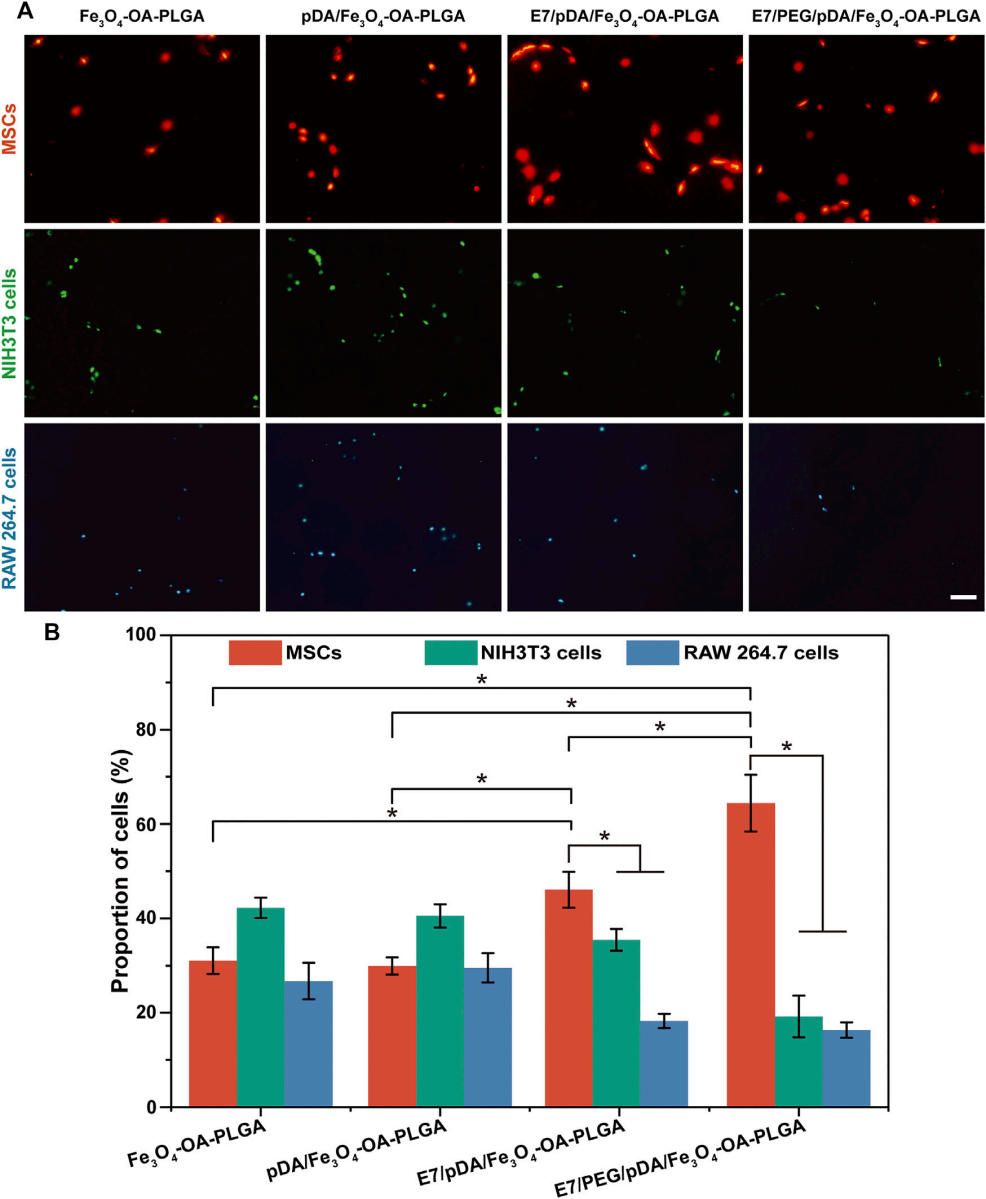
Representative fluorescent images **(A)** of MSCs (DiI, red), NIH3T3 (Calcein-AM, green), and RAW 264.7 cells (Hoechst 33342, blue) adhering to various microspheres after 6 h of separate culture. The proportions **(B)** of adherent MSCs, NIH3T3, and RAW 264.7 cells on the same type of microsphere. Scale bar = 100 μm. At least five fields of view were randomly taken for each group of microspheres. *p* < 0.05, *n* = 3.

The E7 peptide, which was bound on the surface of the microspheres, was initially shown to have a specific affinity for MSCs relative to NIH3T3 (fibroblasts) and RAW 264.7 cells (macrophages). Cell adhesion was detected 6 h after inoculation, as shown in Figure 6. The images revealed that the number of cells adhering to the Fe_3_O_4_-OA-PLGA surface was lower than that on the pDA/Fe_3_O_4_-OA-PLGA surface, regardless of cell type. The addition of dopamine coating on the microspheres increased non-specific cell adhesion, but the cell proportions did not change (Figure 6A). As previously reported (Madhurakkat Perikamana et al., 2015), pDA imparts a positive charge to the material surface, allowing the material to bind more readily to integral protein receptors of the cell membrane, thus improving cell adhesion. On the surface of E7/pDA/Fe_3_O_4_-OA-PLGA, the adhesion of NIH3T3 and RAW 264.7 cells was not higher than that for pDA/Fe_3_O_4_-OA-PLGA, whereas the number of adherent MSCs was increased. On E7/PEG/pDA/Fe_3_O_4_-OA-PLGA microspheres, the cells of MSCs were further increased, while that of NIH3T3 and RAW 264.7 cells were further reduced. Statistical analysis showed that at 6 h post-inoculation (Figure 6B), the proportion of adherent MSCs on the surface of E7/PEG/pDA/Fe_3_O_4_-OA-PLGA was more than two-fold higher than that of NIH3T3 and RAW 264.7 cells. These results indicated that the grafted PEG molecules and E7 peptide effectively resisted the non-specific adhesion of cells.

### 3.7 Selective Adhesion of Co-Cultured Multiple Cells (Static Culture)

**FIGURE 7:**
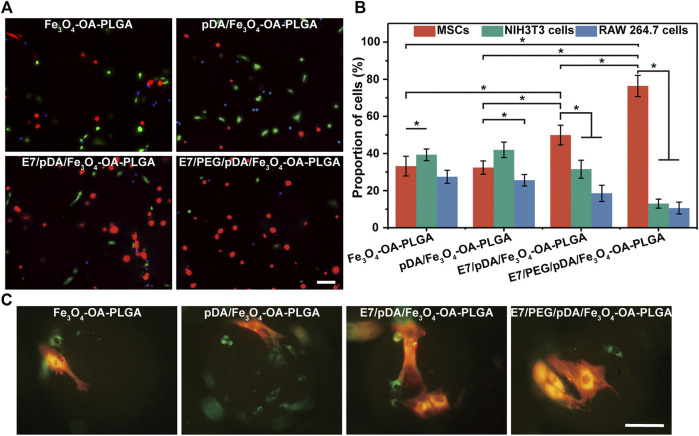
Representative fluorescent images **(A)** of MSCs (DiI, red), NIH3T3 (Calcein-AM, green), and RAW 264.7 cells (Hoechst 33342, blue) co-cultured on different microspheres for 6 h under static conditions. Statistical analysis of the proportions **(B)** of MSCs, NIH3T3, and RAW 264.7 cells per view. The morphology **(C)** of the three types of cells on the microspheres. The red cells were MSCs, and the green cells were NIH3T3 or RAW 264.7 cells. Scale bar = 100 μm. At least five fields of view were randomly taken for each group of microspheres. *p* < 0.05, *n* = 3.

The selective adhesion of MSCs to the E7/PEG/pDA/Fe_3_O_4_-OA-PLGA microspheres was further verified through co-culturing MSCs with NIH3T3 and RAW 264.7 cells under static conditions (Figure 7A). The results showed that the total number of cells on the surface of Fe_3_O_4_-OA-PLGA microspheres was low, and the proportions of the three types of cells were comparable. A large number of adherent MSCs (red), NIH3T3 (green), and RAW 264.7 cells (blue) could be observed on pDA/Fe_3_O_4_-OA-PLGA microspheres. With the addition of E7 peptide, more red cells were observed on the E7/pDA/Fe_3_O_4_-OA-PLGA microspheres, indicating enhanced selectivity for MSCs adhesion. However, E7/PEG/pDA/Fe_3_O_4_-OA-PLGA microspheres exhibited more red cells and fewer green and blue cells than unmodified Fe_3_O_4_-OA-PLGA microspheres. This suggested that the PEG antifouling layer effectively blocked the non-specific adhesion of cells. Accordingly, the proportions of cells on the different microspheres were statistically analyzed (Figure 7B). The percentages of MSCs, NIH3T3, and RAW 264.7 cells cultured on Fe_3_O_4_-OA-PLGA were 33.19 ± 5.32, 39.32 ± 3.11, and 27.49 ± 3.51%, respectively. Similar to separate culture, when cells were co-cultured on the pDA/Fe_3_O_4_-OA-PLGA microspheres, there was no significant change in the proportion of each cell type, although there was an increase in the number of each cell type. With respect to E7/pDA/Fe_3_O_4_-OA-PLGA microspheres, the percentage of MSCs increased from 32.4 ± 3.58% to 49.9 ± 5.27%. Furthermore, on E7/PEG/pDA/Fe_3_O_4_-OA-PLGA microspheres, the percentage of MSCs was increased to 76.39 ± 5.67%. The above results led us to conclude that the selective adhesion of MSCs to E7/PEG/pDA/Fe_3_O_4_-OA-PLGA microspheres was significantly enhanced under static culture conditions. Compared with the situation when the 3 cells were inoculated separately, on the surface of E7/pDA/Fe_3_O_4_-OA-PLGA and E7/PEG/pDA/Fe_3_O_4_-OA-PLGA microspheres grafted with E7 peptide, the proportions of NIH3T3 and RAW 264.7 cells were decreased, which might be due to the increasing selective affinity of microspheres for MSCs, which caused more adherence of MSCs, and competitively reduced the adherence of NIH3T3 and RAW 264.7 cells.

Observing the morphology of the three types of cells on the microspheres (Figure 7C), corresponding to Figure 7A, the E7-grafted microspheres exhibited more adhered MSCs. This also suggests that E7 might influence the subsequent proliferation of MSCs by affecting their early adhesion (Figure 5A).

### 3.8 Selective Adhesion of Co-Cultured Multiple Cells (Dynamic Culture)

**FIGURE 8:**
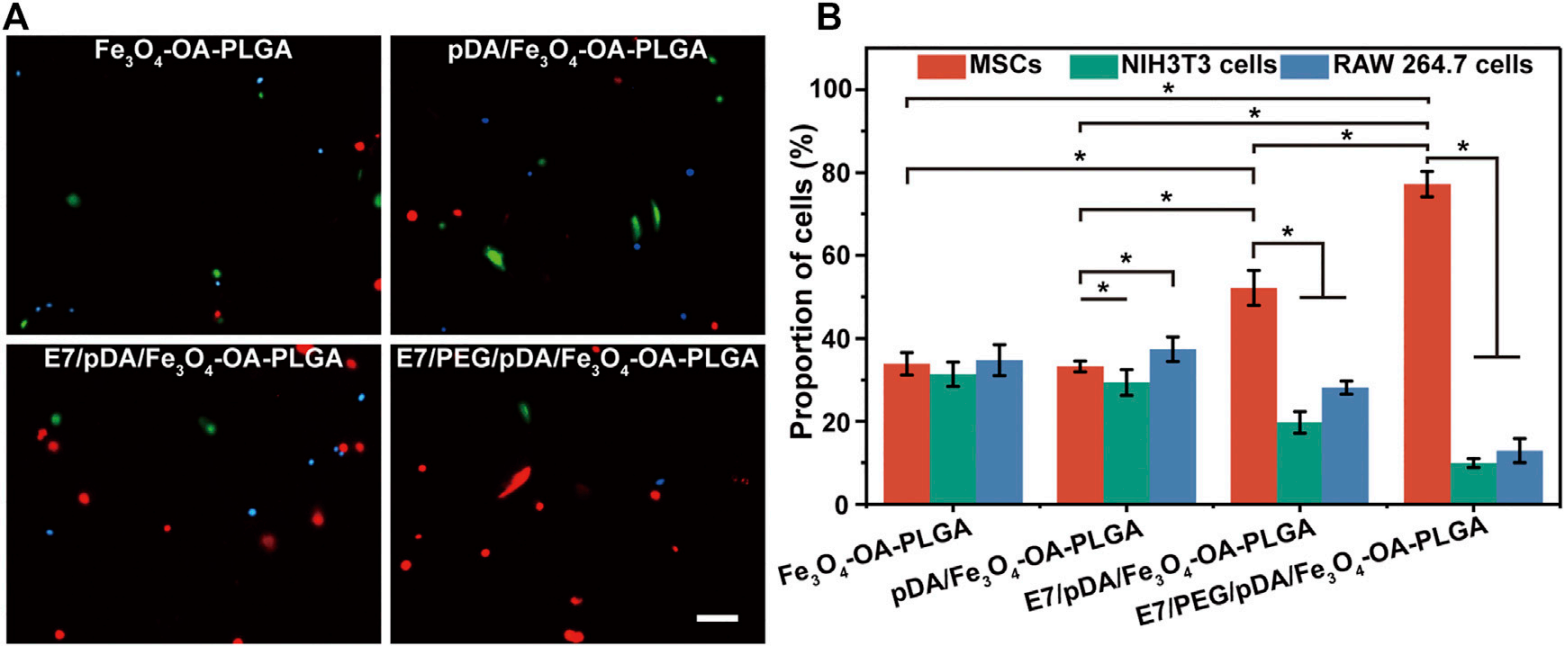
Representative fluorescent images **(A)** of MSCs (DiI, red), NIH3T3 (Calcein-AM, green), and RAW 264.7 cells (Hoechst 33342, blue) co-cultured on different microspheres for 6 h under dynamic conditions. Scale bar = 100 μm. Statistical analysis of the proportions **(B)** of MSCs, NIH3T3, and RAW 264.7 cells per view. At least five fields of view were randomly taken for each group of microspheres. *p* < 0.05, *n* = 3.

Because microspheres are often cultured in a dynamic environment as cell microcarriers, the selective capture of MSCs by the obtained microspheres under dynamic conditions was further investigated (Figure 8A). The total number of adherent cells on the surface of each microsphere decreased compared with that in static culture. Presumably, shear forces in the system may cause adhesion to take longer to achieve (Ning et al., 2013). After modification with dopamine, the number of cells on the pDA/Fe_3_O_4_-OA-PLGA microspheres increased (versus the Fe_3_O_4_-OA-PLGA microspheres group). Unsurprisingly, modification of the E7 peptide resulted in more MSC adhesion, as under static culture conditions. On the E7/PEG/pDA/Fe_3_O_4_-OA-PLGA microspheres, the adherent numbers of NIH3T3 and RAW 264.7 cells were also decreased. These results showed that the PEG antifouling layer could also inhibit non-specific cell adhesion under dynamic conditions. Accordingly, statistical analysis of cell proportions was performed (Figure 8B). The percentages of MSCs, NIH3T3, and RAW 264.7 cells cultured on Fe_3_O_4_-OA-PLGA microspheres were 34.76 ± 3.77, 31.35 ± 2.94, and 33.893 ± 2.71%, respectively. On pDA/Fe_3_O_4_-OA-PLGA microspheres, the proportions of cells were 37.37 ± 2.94, 29.36 ± 3.10, and 33.27 ± 1.29%. For E7/pDA/Fe_3_O_4_-OA-PLGA microspheres, MSCs increased to 52.16 ± 4.22%, while NIH3T3 and RAW 264.7 decreased to 19.75 ± 2.59% and 28.09 ± 1.58%. The proportion of MSCs on E7/PEG/pDA/Fe_3_O_4_-OA-PLGA microspheres further increased to 77.19 ± 3.05%. The results showed that the E7/PEG/pDA/Fe_3_O_4_-OA-PLGA microspheres were equally selective for MSCs under dynamic conditions compared with that in static culture.

### 3.9 Stemness of the Adhered MSCs

**FIGURE 9:**
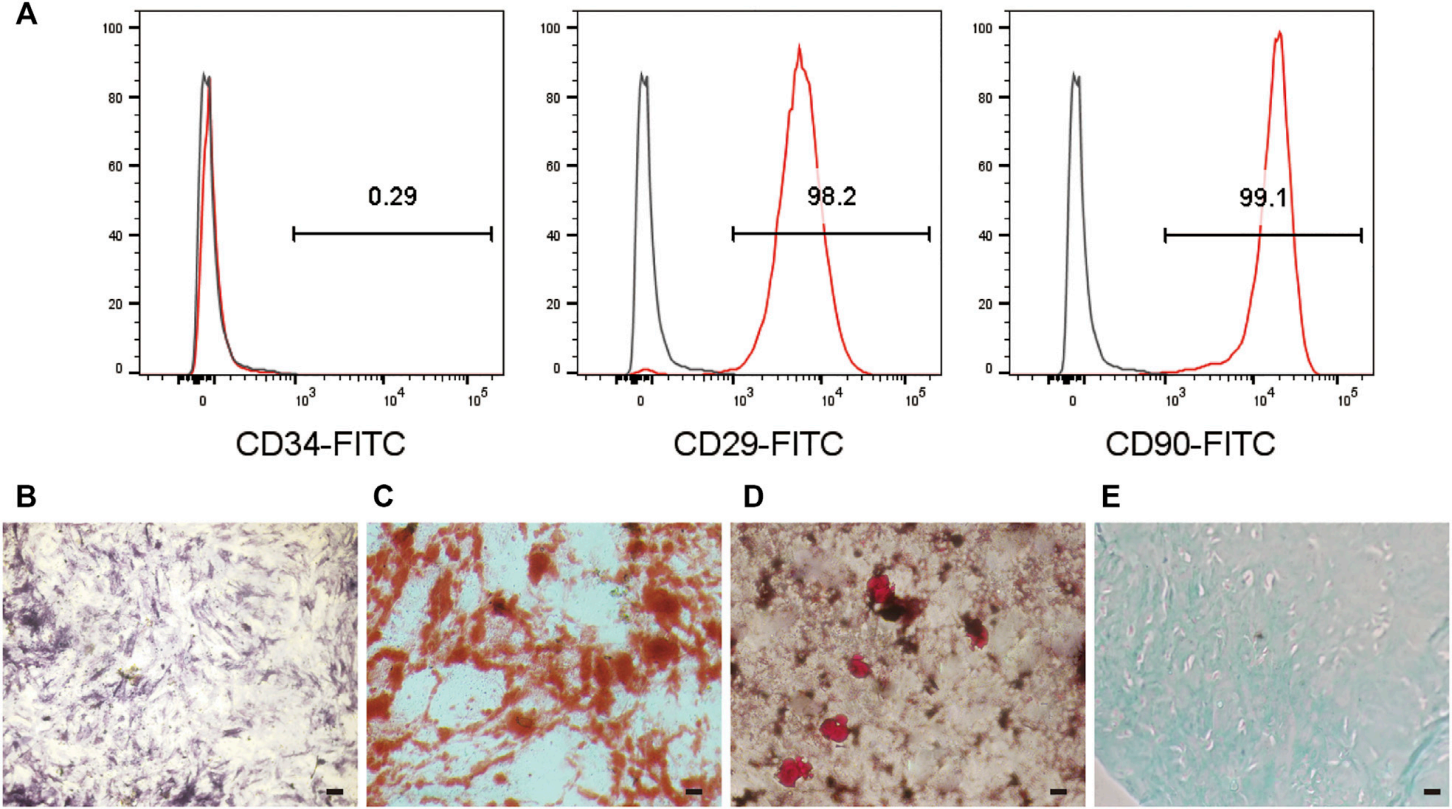
Flow cytometric analysis **(A)** of MSCs cultured on the E7/PEG/pDA/Fe_3_O_4_-OA-PLGA microspheres for 7 days. ALP **(B)** and ARS staining **(C)** after osteogenesis induction for 7 or 14 days in MSCs pre-seeded on the E7/PEG/pDA/Fe_3_O_4_-OA-PLGA microspheres. Oil red O staining **(D)** after lipogenesis induction for 21 days in MSCs that were pre-seeded on the E7/PEG/pDA/Fe_3_O_4_-OA-PLGA microspheres. Alcian blue staining **(E)** after chondrogenic induction for 28 days in MSCs pre-seeded on the E7/PEG/pDA/Fe_3_O_4_-OA-PLGA microspheres. Scale bar = 100 μm.

MSCs can offer promising therapeutic potential for many diseases (Patel et al., 2013). Because of the limited fraction of MSCs collected from tissues, *in vitro* expansion of MSCs is required; nevertheless, loss of stemness and undesired differentiation of MSCs during *in vitro* culture can diminish their efficiency (Saei Arezoumand et al., 2017). Therefore, their stemness qualities must be preserved *in vitro* (McKee et al., 2017). To investigate the stemness retention and differentiation potential of MSCs expanded on the surface of E7/PEG/pDA/Fe_3_O_4_-OA-PLGA microspheres, the cells were inoculated for an additional 7 days and harvested; then, flow cytometric analysis and induced differentiation were carried out (Figure 9). The positive expression indicators CD29 and CD90 and the negative expression indicator CD34 by MSCs were taken as identification reference indicators. Flow cytometry (Figure 9A) showed that the cells did not express CD34, but did express CD29 and CD90. This was consistent with the surface marker characteristics of MSCs (Harting et al., 2008). ALP activity was a sign of early osteogenic differentiation in MSCs (Weinreb et al., 1990). Figure 9B showed positive ALP staining. Mineral deposition has been shown to be a late marker of osteogenesis, and calcium deposition can be measured using alizarin red staining (Liu et al., 2016). Significant calcium deposition (Figure 9C) was found in MSCs inoculated on E7/PEG/pDA/Fe_3_O_4_-OA-PLGA microspheres. With regard to lipogenic differentiation, oil red O staining (Figure 9D) showed the existence of lipid vacuoles. Alcian blue staining (Figure 9E) confirmed chondrogenic morphogenesis. The results demonstrated that cells growing on the microspheres still retained the ability to differentiate. Furthermore, we found that the number of MSCs captured on E7/PEG/pDA/Fe_3_O_4_-OA-PLGA under dynamic conditions was statistically significantly lower than the number of MSCs captured under static conditions (Supplementary Table S1). Consistent with the preceding results, it is possible that shear forces affected early cell adhesion (Ning et al., 2013), and such shear forces might, to some extent, also mimic the actual stress environment *in vivo* (Zhang et al., 2010).

Additionally, microspheres can be used not only *in vitro*, but also *in vivo* as supporting material and carriers for cell growth and delivery (Hiraoka et al., 2006; Kang et al., 2009; Kamali et al., 2019). Nevertheless, the non-specific capture of fibrocytes and inflammation-related cells may lead to fibrosis because of the complexity and diversity of the cells participating in the tissue repair procedure (Tabata, 2009). Hence, in order to achieve tissue regeneration without fibrosis, selective capture of MSCs by the E7-modified microspheres in the presence of fibrocytes and inflammatory cells is crucial. MSCs can selectively adhere to such microspheres, reducing the potential for fibrosis and inflammation caused by fibroblasts and inflammatory cells.

## 4 Conclusion

In this study, E7 peptide was grafted onto the surface of PLGA magnetic microspheres via PEG for MSC sorting by selective adhesion. The superparamagnetism of Fe_3_O_4_-OA NPs was verified by TGA/DTA, VSM, and TEM characterization. Observations from light microscopy and electron microscopy demonstrated that the microspheres had uniform size and satisfied surface morphology requirements. Grafting of PEG exhibited anti-cell adhesion ability, and the subsequent E7 peptide modification fulfilled cell adhesion requirements. The separate culture and co-culture of multiple types of cells on the peptide-modified microspheres, both in static and dynamic conditions, revealed efficient cell sorting ability. Therefore, we demonstrate here that the PLGA magnetic microspheres grafted with E7 peptide via PEG represent a promising platform for MSC sorting and expansion. The method of preparation of microspheres in this study is simple, and the microspheres are easy to use at a low cost. It is noteworthy that, at near 80%, the sorting efficiency of the microspheres is not as high as that of classical sorting methods using antibodies. This might be related to the grafting amount of the peptide, which is also to be improved in our subsequent work.

## Data Availability

The original contributions presented in the study are included in the article/Supplementary Material, further inquiries can be directed to the corresponding authors.
