# Supplementary material for: Peptide-Grafted Microspheres for Mesenchymal Stem Cell Sorting and Expansion by Selective Adhesion
Source: Front Bioeng Biotechnol. 2022 Apr 12;10:873125. doi: 10.3389/fbioe.2022.873125 (PMC9039221; doi:10.3389/fbioe.2022.873125)
Supplement: Supplementary file 1 [file DataSheet1.docx]

Supplementary Material

# Supplementary experiment

## Dispersibility

Fe_3_O_4_-OA NPs were dispersed in chloroform at a final concentration of 10 mg mL^-1^ and placed in cuvettes for 50 h at room temperature without moving, and photographs were taken at that time point. The dispersibility and stability of the NPs in chloroform could be judged according to the precipitation of the solution.

## Cytotoxicity test

0.1 g of microspheres were submerged in 1 ml of Dulbecco's modified Eagle medium (DMEM): F12 and shaken at 150 rpm for 24 h at 37 °C. The medium extract was sterilized using a syringe filter device (Millex-GP, 0.22 μm, Millipore). MSCs cells were plated on 48-well plates at a density of 2×10^4^ cells well^-1^ and incubated for 24 h to adhere. The medium was then replaced with new medium (control), 100% (v/v) extract and 50% (v/v) extract (diluted with DMEM). After a further 24 h of incubation, 50 μL of CCK-8 reagent was added to each well and incubated at 37 °C for 2 h. Following incubation, the solution (100 μL) in each well was pipetted to another 96-well plate and detected at 450 nm with a microplate reader (Infinite M 200, Tecan, Switzerland). Cell viability (%) = OD values (Samples)/OD values (Controls) ×100 was used to calculate the cytotoxicity of the samples. The final results were derived using the average value of three parallel samples.

# Supplementary Figures and Tables


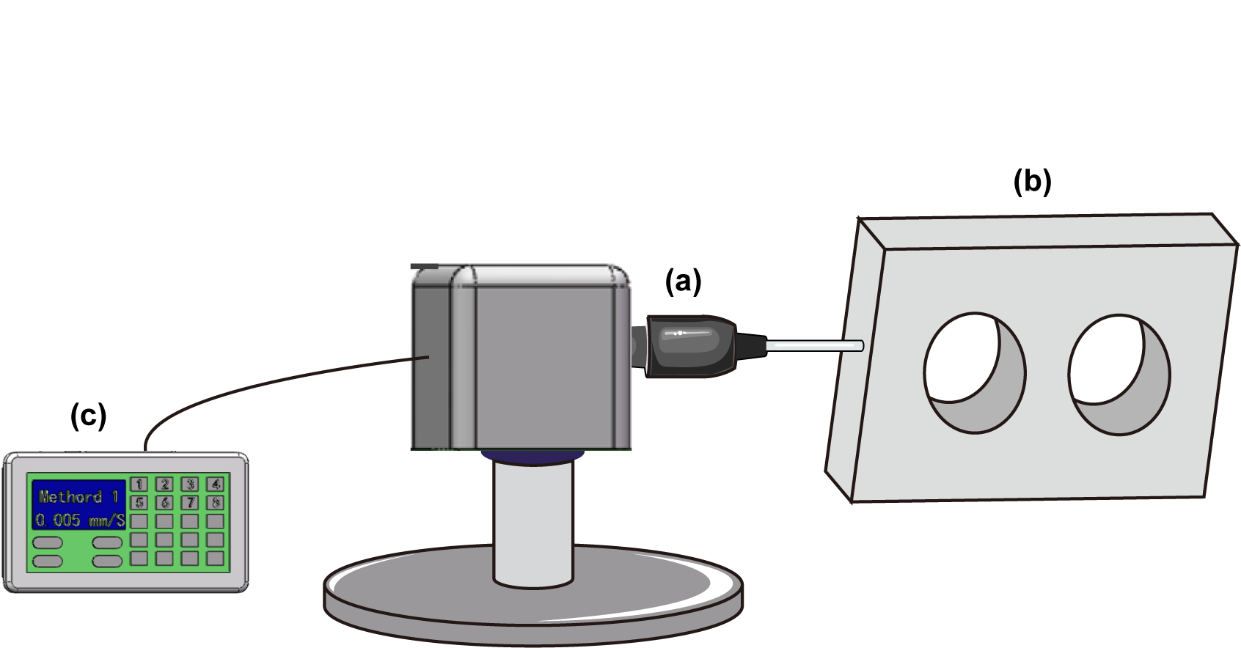


**Supplementary Figure 1.** Schematic diagram of the home-made equipment used for dynamic culture. When the power is turned on, **(a)** rotates to drive **(b)**, and **(c)** is the control component to set the program to be executed.

**
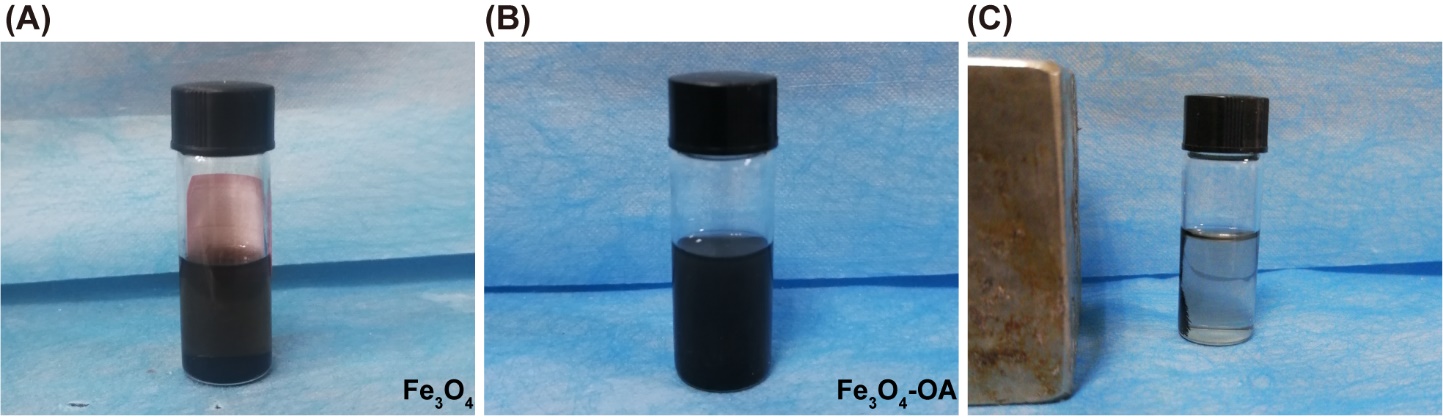
**

**Supplementary Figure 2.** Images of Fe_3_O_4_ **(A)** and Fe_3_O_4_-OA **(B)** NPs in chloroform after standing for 50 h. **(C)** Responsiveness of Fe_3_O_4_-OA NPs in the presence of a magnet.

**
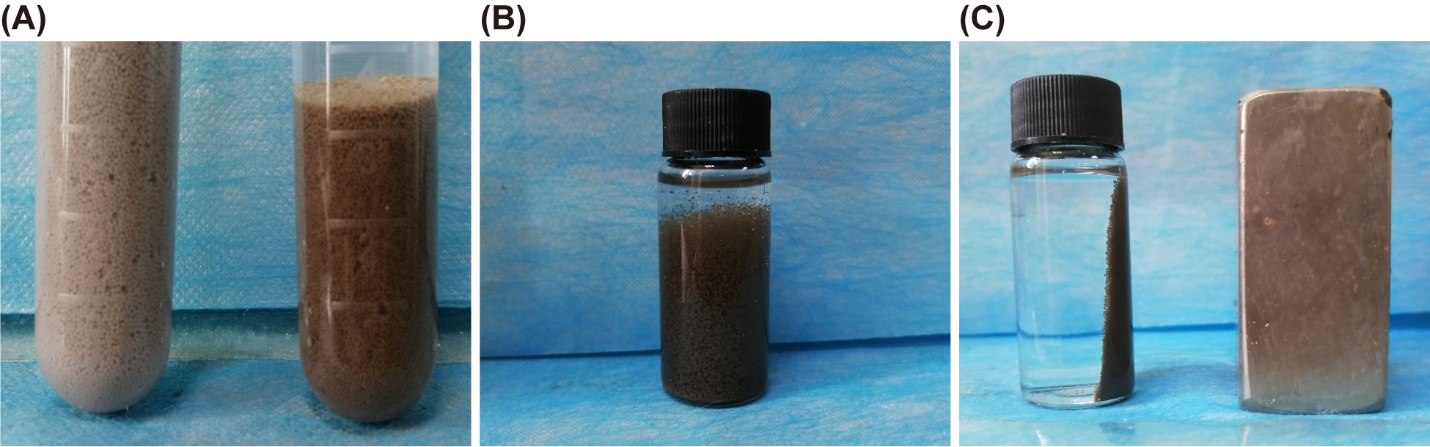
**

**Supplementary Figure 3.** Gross observation **(A)** of Fe3O4-PLGA (left) and Fe3O4-OA-PLGA microspheres (right). Microsphere suspensions without **(B)** or with **(C)** a magnet.

**
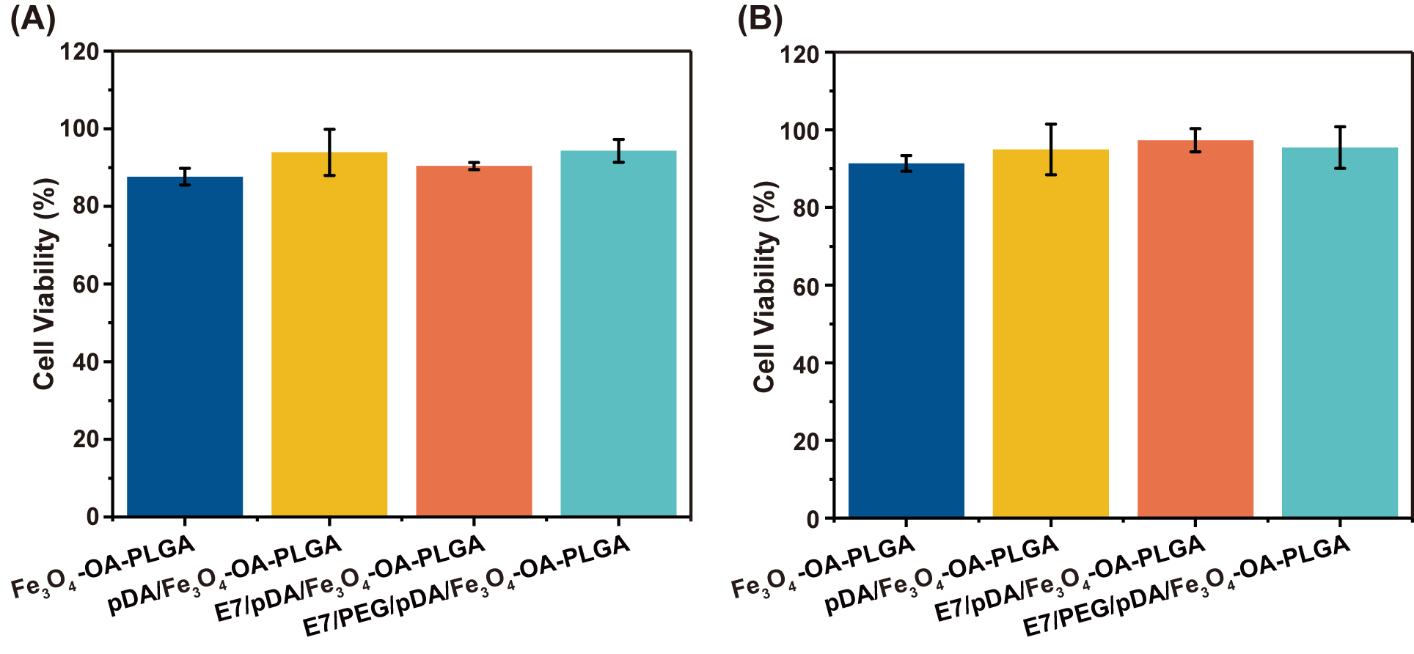
**

**Supplementary Figure 4.** Cell viability of MSC incubated in 100% extraction medium **(A)** and 50% extraction medium **(B)** for 24 hours.

**Supplementary Table 1.** Cell count data were analyzed with t-test between static culture and dynamic culture groups.

| Group | Cell Count/pic | t value | P value |
| --- | --- | --- | --- |
| Static culture group | 31.4±3.94244 | 0.05 | 5.43517×10^-14^ |
| Dynamic culture group | 13.46667±2.92445 |  |  |

P<0.05, there was a significant difference between the two groups of data.
